# War-related eye trauma: a study of civilian and military cases from Ukraine's ongoing conflict

**DOI:** 10.3389/fpubh.2025.1489445

**Published:** 2025-02-10

**Authors:** Kamil Jonak, Magdalena Matysiak, Tomasz Choragiewicz, Dominika Nowakowska, Andriy Zimenkovsky, Volodymyr Shybinskyi, Myroslawa Sekh, Robert Karpiński, Arkadiusz Podkowiński, Robert Rejdak

**Affiliations:** ^1^Department of Technical Informatics, Lublin University of Technology, Lublin, Poland; ^2^Chair and Department of General and Pediatric Ophthalmology, Medical University of Lublin, Lublin, Poland; ^3^Department of Health Care Management, Pharmacotherapy & Clinical Pharmacy, Danylo Halicky Lvov National Medical University, Lviv, Ukraine; ^4^Dental Medical Centre, Danylo Halicky Lviv National Medical University, Lviv, Ukraine; ^5^Department of Machine Design and Mechatronics, Faculty of Mechanical Engineering, Lublin University of Technology, Lublin, Poland; ^6^Institute of Medical Sciences, The John Paul II Catholic University of Lublin, Lublin, Poland; ^7^1st Department of Psychiatry, Psychotherapy and Early Intervention, Medical University of Lublin, Lublin, Poland; ^8^Da Vinci NeuroClinic, Lublin, Poland

**Keywords:** ophthalmology, war in the Ukraine, epidemiology, eye injuries, trauma, eye

## Abstract

Forecasts indicate a substantial increase in the occurrence of eye injuries in future armed conflicts. The Russian invasion of Ukraine, launched in February 2022, caused numerous eye injuries among civilians as well as military personnel, generating a serious epidemiological threat related to vision loss. The main goal of this study was to analyze different eye traumas in the Ukrainian population caused by hostilities, which could allow for a relative estimate of the occurrence of long-term consequences for the health care system, such as loss of vision in a large group of citizens. To assess the scale and types of eye injuries, we analyzed around 500 eye images from 470 patients who were selected because they had sustained eye injuries and were treated at a single hospital. The findings reveal that the most prevalent types of injuries were macular disorders, accounting for 49% of cases, retinal vascular changes at 30.2%, and optic nerve disorders at 22.4%. Additionally, we observed different percentages of eye injuries in the military personnel group compared to civilians. These results highlight the significant impact of eye injuries caused by war operations on the health care system. However, further research and collaborative efforts are needed to fully assess the epidemiological burden and to inform the development of effective treatment and prevention strategies.

## 1 Introduction

The historical trajectory of humanity has been profoundly intertwined with the phenomenon of warfare. Throughout the ages, there has been a continuous enhancement in methods devised for inflicting harm upon others. This escalation was paralleled by advancements in the field of medicine, which endeavored, with varying degrees of success, to provide healing for those afflicted by the progressively more lethal innovations. As warfare evolved over the centuries, there was a notable transformation in the nature of injuries, their locations, and most critically, their mortality rates. Notably, the latter witnessed a significant reduction, particularly in the latter half of the 19^th^ century. Entering the 20^th^ century, this trend in mortality rates has remained relatively unchanged, and it appears highly probable that this pattern will persist (1).

Projections suggest a significant surge in the incidence of eye injuries in future armed conflicts. The 20^th^ century witnessed a substantial rise in the number of individuals harmed in military engagements, with civilian casualties constituting as much as 90% of total casualties in global military conflicts. Fragmentation weapons have been identified as the primary contributor to the escalation in ocular injuries. Since World War II, there has also been a notable increase in the number of civilian patients receiving treatment in U.S. military field hospitals stationed abroad (2, 3).

Renowned military ophthalmologist, Dr. Albert Hornblass, following his tenure at the 71^st^ Evacuation Hospital in Pleiku, Vietnam, conducted extensive research on the prevalence of eye injuries in both American and international military conflicts. His studies revealed that during the American Civil War, eye injuries accounted for 0.5% of all casualties, a figure that escalated to between 5% and 9% during the Vietnam War. This rate further increased to 13% during the operations in Desert Shield and Desert Storm in Kuwait (4).

Eye injuries during armed conflicts are common and can stem from various causes. The most frequent types of eye injuries include mechanical, thermal, and chemical trauma, injuries related to radiation, blast injuries, laser injuries, as well as stress-related injuries. Mechanical injuries are caused by direct impact, for instance, from fragments, metal pieces, stones, or even wind and dust (5–7). These can lead to severe damages such as eyeball perforation, lens dislocation, or corneal ruptures. Thermal and chemical injuries may result from explosions, exposure to corrosive substances, or combat gases. They can lead to burns of the eyelids, conjunctiva, cornea, and deeper ocular damages (8, 9). High exposure to radiation, such as during a nuclear blast, can damage the retina and other ocular structures (10). Explosions can generate shockwaves, which can cause eye trauma without direct contact with a foreign object (11). In warfare, exposure to intense laser light sources can cause serious retinal damage (12, 13). The impact of war-related stress on eye health may include vision problems caused by psychological factors, such as post-traumatic stress disorder (14). Some of listed possible eye injuries are easier to threat but most of them cause irreversible loss of vision. Thus, in a long-term perspective, eye injuries caused by hostilities or related events have a substantial impact on the public health sector (15).

On February 24, 2022, Russia initiated a comprehensive military incursion into Ukraine. Prior to this conflict, Ukraine's healthcare system was not particularly distinguished, grappling with challenges such as frequent interruptions in medication supply for chronic conditions, complaints from medical professionals regarding inadequate remuneration, and widespread extortion within hospital settings. According to The International Agency for the Prevention of Blindness report, before the war there were 2973 ophthalmologists in Ukraine (6.6 per 100,000 population) and around 2.1 million people classified as blind. Nevertheless, as part of their strategic operations, the Russian forces commenced assaults on healthcare facilities (290 attacks through June 9, 2022), destroying Ukrainian medical potential and leaving hundreds of people without healthcare (16).

Although the conflict has been ongoing for over 2 years, comprehensive information regarding the extent of eye trauma sustained in the war in Ukraine remains limited. Some historical data suggest an upward trend in eye trauma prevalence, although the data sources require cautious interpretation. For example, references indicate that the frequency of eye traumas in Ukrainian conflicts has ranged from 0.65% during the Crimean War in the 1850s to 7.0%−14.0% during the Anti-Terrorist Operation in eastern Ukraine in 2014 (17, 18).

Despite limited research on Ukraine conflict-related eye injuries, existing reports shed light on this critical issue. The International Agency for the Prevention of Blindness highlights widespread vision problems and barriers to accessing eye care (19). Meanwhile, the World Health Organization documents attacks on health facilities, exacerbating the health crisis and complicating the treatment of ocular trauma (20). Emerging studies reveal severe injuries from cluster munitions, which can lead to irreversible vision loss if untreated (21). The high prevalence of blast-related eye injuries in contemporary warfare underscores the need for protective measures, as enforced eye protection reduces incidence and severity (22). These findings underscore the urgent need for comprehensive, evidence-based investigations into eye trauma in the current Ukrainian conflict to guide prevention and treatment. Continued monitoring, data collection, and analysis are vital to understand the scope of ocular injuries and develop effective strategies to minimize vision loss and improve patient outcomes.

This study provides a detailed analysis of eye injuries in individuals affected by the ongoing conflict in Ukraine, focusing on military personnel and civilians who sustained war-related ocular trauma. However, it is important to note that this study is based on a case series from a single hospital, and as such, the findings cannot be generalized to the broader Ukrainian population. The patients included in the study were predominantly individuals who presented with acute war-related injuries and had not previously reported deteriorating vision. Therefore, the results of this study primarily reflect the types and severity of eye injuries caused directly by the ongoing conflict, rather than a broader assessment of the general population's ocular health. Further research is needed to determine whether these findings can be extended to the wider Ukrainian population, including those who may not have sought medical care or those who experienced less severe, non-traumatic vision impairments.

## 2 Materials and methods

### 2.1 Participants, inclusion, and exclusion criteria

The recruitment of participants was conducted from January 2023 to June 2024 and included both civilian and military personnel. It is important to note that this study does not exclusively focus on civilians. Participants were recruited from the Lviv Regional Clinical Hospital, located at Chernihivska Street, Number 7, Lviv, Lvivska Oblast, 79010. This facility provides medical services to both civilian and military patients, ensuring comprehensive care for individuals with traumatic eye injuries regardless of their status. Ophthalmologists with expertise in diagnosing and managing traumatic eye injuries enrolled 470 participants.

All patients provided informed consent, and the study protocol was approved by the Bioethical Commission of the Medical University of Lublin. Furthermore, all methods used in the study adhered to relevant guidelines and regulations established by the Bioethical Commission.

#### 2.1.1 Criteria for patient inclusion

- Individuals who had eye traumas caused by hostilities (i.e., bombardment, filed combat etc.) and were in a general state of health that permits thorough documentation of the eye globe.
- Provision of informed consent for participation in the study.

#### 2.1.2 Criteria for exclusion

- Absence of a signed informed consent for study participation.
- Presence of a risk that participation in the study might lead to life-threatening conditions or worsen the general state of health.

### 2.2 Examination protocol

A proficient ophthalmic technician captured eye fundus images, including those centered on the papilla and macula. This was accomplished using both tabletop and handheld Optomed Aurora fundus cameras (manufactured by Optomed, located in Oulu, Finland). The process was uniformly conducted with pupil dilation using 1% tropicamide to ensure consistent image quality. The portable fundus camera was employed to acquire images with a 50-degree field of view and a resolution of 5 megapixels. Subsequently, these images were archived in JPEG format (Joint Photographic Experts Group).

Qualified ophthalmologists conducted an analysis of various aspects pertaining to the quality of the captured images. Each quality parameter were assigned a score, ranging from 2 (excellent) to 0 (poor or ungradable). An image were classified as superior if it exhibits well-centered features of the macula or optic disc, clear visibility of fundus vessels, and discernible retinopathy along with the entirety of the image being visible.

An image were considered as satisfactory if the macula or optic disc was centered, the fundus vessels and any retinopathy were distinguishable, and more than 80 percent of the image was visible. Conversely, an image was considered ungradable if it was not centered, blurred to the extent that retinal vessels or characteristics of retinopathy were undetectable, or if <80 percent of the image was visible.

Clinical observations and characteristics of eye lesions were thoroughly evaluated, encompassing the distinction between open and closed injuries, the presence of intraocular foreign bodies, and the extent of damage to ocular structures such as the eyelids, cornea, iris, lens, vitreous body, retina, retinal vessels, and optic disc, along with the presence of retinal detachment or endophthalmitis.

Visual acuity of the patient were quantified with a decimal scale and juxtaposed with the established Eye Trauma Score. All patient data were anonymized to ensure confidentiality. The transfer of this data were conducted in strict compliance with both Polish (including RODO) and Ukrainian laws governing personal data protection. The demographic information of the patient, encompassing age, gender, occupation, and social status, were also analyzed.

A proficient statistician made statistical analysis utilizing the STATISTICA 13.3 software package (StatSoft Inc., Tulsa, OK, USA). For discrete outcome variables, descriptive statistics were delineated as frequencies and proportions (*n*, %).

## 3 Results

The average as well as median age of the participants was approximately 46 years. The minimum age of participants was 16 years, while the maximum age was 85 years, reflecting a wide range of ages within the sample. The standard deviation of age was approximately 18 years, indicating significant variation in age among the participants.

Men constituted a significant majority of the study sample, totaling 79% individuals. Women were represented in a smaller number, amounting to 21% of individuals. The largest subgroup in the study comprised military personnel, with a count of 52% individuals, making it the largest group among the participants.

**Figure 1:**
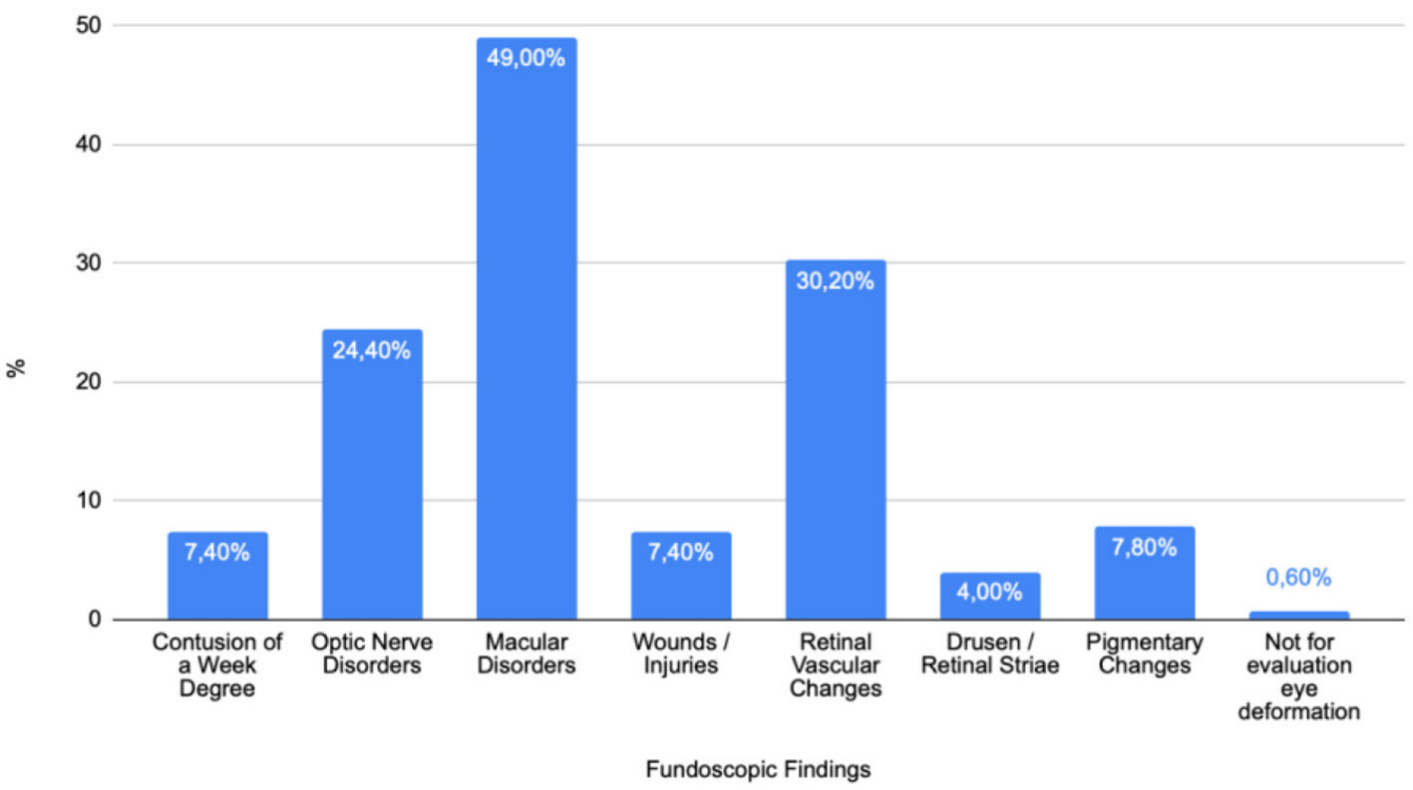
Fundoscopic findings in ukrainian war patients: an analysis of ocular pathologies.

The dataset contains 500 eye images from 470 patients, categorized into various ocular conditions (Figure 1). The first category, “Contusion of a Mild Degree,” includes 37 eyes, representing 7.4% of the total. These cases involve superficial injuries such as eyelid and conjunctiva lesions or corneal erosion, where the fundus of the eye remains unaffected, with no pathological changes observed. The second category, “Optic Nerve Disorders,” encompasses 122 eyes, or 22.4% of the cases, involving a range of issues affecting the optic nerve, which can impact vision quality and acuity. The largest category, “Macular Disorders,” includes 245 eyes, accounting for 49.0% of the total. These disorders affect the central part of the retina, the macula, which is crucial for sharp, central vision.

Another significant category is “Serious Trauma,” involving 37 eyes or 7.4% of the total, and includes more severe injuries such as retinal hemorrhages and choroidal breaks that can affect various parts of the eye, potentially leading to lasting damage or impaired vision. “Retinal Vascular Changes” occurred in 151 eyes, making up 30.2% of the cases, and involve changes in the blood vessels of the retina, which can be indicative of systemic health issues like hypertension or diabetes. The “Drusen/Retinal Striae” category, involving 20 eyes or 4.0%, refers to the presence of drusen or retinal striae, which can be associated with aging and macular degeneration, indicating structural changes in the retina. “Pigmentary Changes” were observed in 39 eyes, or 7.8%, and involve alterations in the retinal pigment epithelium, potentially indicating various retinal disorders that can affect vision.

Finally, the “Not for Evaluation” category includes 3 eyes, representing 0.6% of the total, which were too damaged to allow for a proper examination of the fundus. The severity of these injuries precluded a complete assessment, making it impossible to determine the extent of damage or underlying conditions. This data highlights the diversity and complexity of eye injuries and conditions, emphasizing the need for comprehensive ocular assessments in trauma cases.

**Figure 2:**
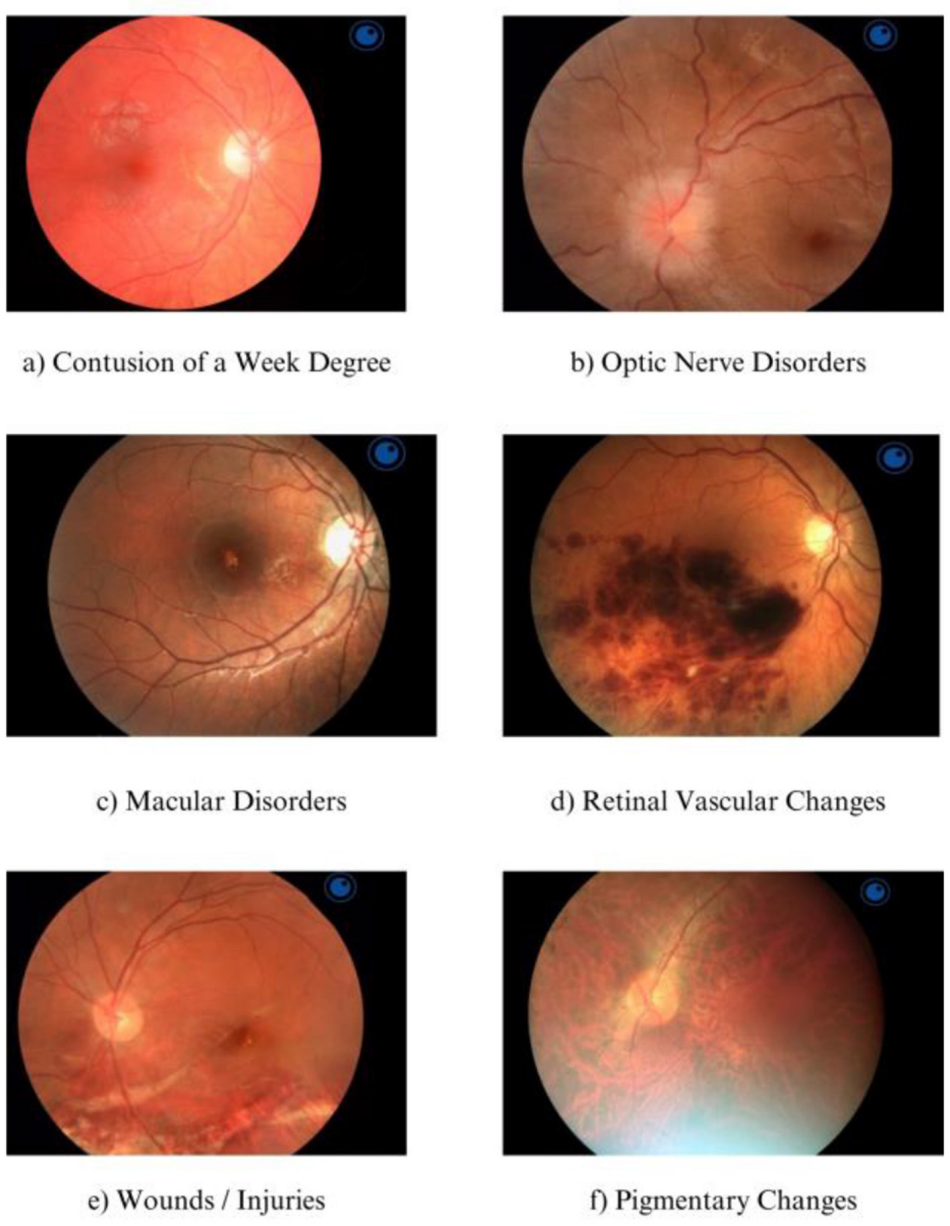
Sample fundus photographs of analyzed patients. **(A)** healthy eye with only minimal bruising, indicating a mild injury. **(B)** Shows a patient with swelling of the optic nerve head, suggesting a more severe injury that could impact visual function. **(C)** Illustrates a case of a macular hole, a significant damage that may lead to substantial loss of visual acuity. **(D)** Presents a hemorrhage, likely resulting from mechanical trauma, highlighting the need for immediate medical intervention. **(E)** Injury caused by a foreign body in the eye, which requires prompt action to prevent further damage. **(F)** Pigmentary changes, which could be due to long-term effects of the injury or other pathological processes. The article emphasizes the importance of prompt diagnosis and appropriate treatment to prevent serious health complications associated with ocular injuries.

In most cases, a single eye could qualify for multiple categories of injury. Frequently, macular disorders, retinal vascular changes, and optic nerve disorders were reported for the same eye. In such instances, one eye could appear in up to three categories simultaneously due to the extensive nature of the injury. Patients with pre-existing ocular conditions experienced exacerbation of their diseases as a result of the post-traumatic injuries. Samples of injuries were presented on Figure 2.

**Figure 3:**
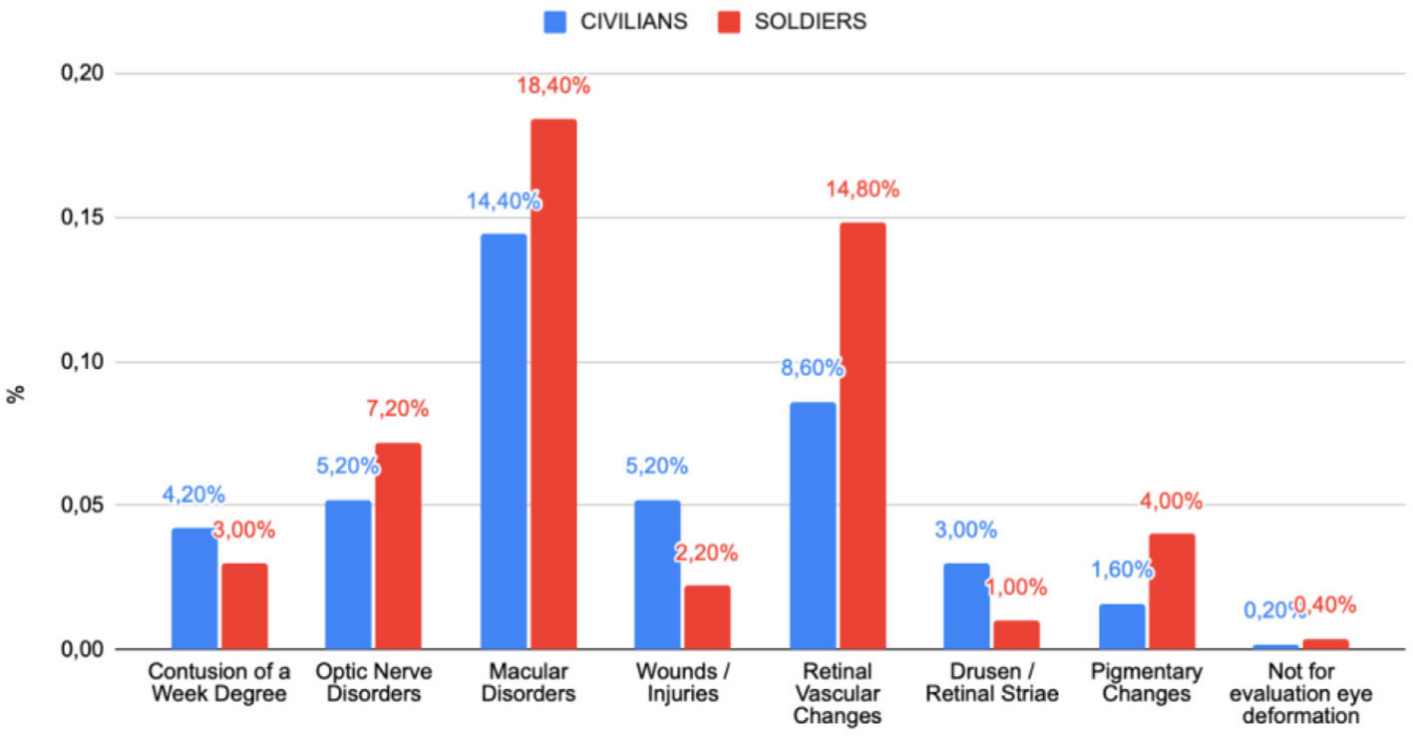
Comparison of pathological changes in the fundus of the eye between military personnel and civilians.

The comparative analysis of fundoscopic findings among war patients in Ukraine includes both military personnel and civilians, revealed a range of ocular pathologies resulting from the conflict (Figure 3). The data highlight several key differences in the prevalence of specific conditions between these two groups.

Among military personnel, macular disorders were the most prevalent pathology, affecting 18.40% of the group, indicating a significant impact of combat conditions on eye health. Retinal vascular changes were also common (14.8%), potentially associated with intense stress or mechanical trauma. Additionally, optic nerve disorders (7.2%) and pigmentary changes (4.00%) occurred more frequently among military personnel, suggesting prolonged exposure to harmful environmental and physical factors. Less common findings include minor contusions (3.00%) and eye wounds/injuries (2.2%).

Among Civilians, though were also exposed to war-related injuries, different patterns of pathology also were observed. Macular disorders were also the most common condition among civilians (14.4%), though with a slightly lower prevalence than among military personnel. Eye wounds and injuries account for 5.2% of cases, a higher percentage than among military personnel, possibly indicating different types of exposure. Optic nerve disorders (5.2%) and retinal vascular changes (8.6%) were also presented, though less frequently than in the military group. Other conditions, such as drusen/retinal striae (3.00%) and pigmentary changes (1.6%), were less common.

## 4 Discussion

One of the main goals of the presented study was to describe the distribution of various types of eye injuries in the Ukrainian population resulting from armed conflict. The findings indicate that this objective has been successfully met, with a thorough and detailed analysis of the frequency and nature of ocular injuries. The study highlights that macular disorders were the most common, accounting for 49% of cases, followed by retinal vascular changes at 30.2% and optic nerve disorders at 22.4%. This descriptive analysis provides valuable information to guide future medical interventions and treatment strategies.

A better understanding of the structure, frequency, and types of eye injuries resulting from warfare is critical to improving health system performance. An analysis of lessons learned from the conflicts in Iraq and Afghanistan (23) indicates that accurate data on injury characteristics can lead to more efficient redistribution and strategic deployment of specialized equipment in medical facilities. In addition, the introduction of ongoing consultations with experts in the field of ocular traumatology and the development and application of technologies such as robotic microsurgery can significantly improve treatment outcomes (24). Experience from the global war on terror shows that a systematic approach to treating ocular injuries on the battlefield not only reduces the long-term health effects on patients, but also eases the burden on the healthcare system by optimizing diagnostic and therapeutic processes (24).

When comparing the results presented in this study with those from other research, several notable similarities and differences emerge. Studies by Hornblass and other research on wartime injuries (2, 4) indicate a rising percentage of eye injuries across various conflicts, aligning with the upward trend observed in the current study. These results suggest that the risk of eye injuries significantly increases during armed conflicts, consistent with predictions outlined in the literature (1). Hornblass's research shows that eye injuries during World War I were 0.5%, rising to 13% during the Vietnam War and subsequent conflicts. The Ukrainian study, which also reported a 13% incidence of eye injuries, encompasses a broader range of damage, potentially reflecting increased conflict intensity and the use of advanced military technologies such as fragmentation weapons and lasers (11, 12).

The study results indicate that the predominant types of eye injuries were macular disorders and retinal vascular changes. In the existing literature, such findings are frequently associated with high exposure to mechanical and thermal injuries, typical of armed conflicts (5, 8). Ukrainian study also identified injuries related to foreign bodies and pigmentary changes in the retina, suggesting possible long-term effects of trauma or inadequate initial treatment.

Additionally, comparing the findings of our study with previous analyses of ocular injuries in wartime conditions, we observe common trends and new challenges associated with advanced weaponry and demographic shifts among casualties (25, 26). For instance, similar to the increase in intraocular foreign body injuries during the Iran-Iraq War, our study also reports a rise in complex injuries resulting from modern explosive devices, such as improvised explosive devices (IEDs). In both cases, the implementation of comprehensive treatment strategies that account for concurrent injuries, like traumatic brain injuries often accompanying severe ocular trauma, is crucial (27).

Literature, such as the analysis of ocular trauma in asymmetric conflict and proxy war environments, highlights that factors like age-related changes and pre-existing conditions can significantly impact the management of ocular injuries (28). For example, in our study, older patients with pre-existing conditions, such as diabetes or hypertension, required specialized approaches in both anesthesia selection and long-term rehabilitation planning, aligning with findings from other studies discussing the complexities of treatment in such cases (24, 29–31).

Moreover, differences in the incidence of injuries between military personnel and civilians suggest distinct exposure patterns to risk factors associated with conflict. Military personnel are more frequently affected by macular disorders and retinal vascular changes, which may be attributed to intense stress or mechanical trauma. Civilians, while also exposed to conflict-related injuries, exhibit different patterns of pathology, suggesting alternative types of exposure.

In the context of eye injury treatment in war settings, research underscores the importance of prompt intervention and appropriate surgical techniques to preserve vision (8, 11). The findings from the Ukrainian study confirm the critical need for early diagnosis and intervention, particularly for retinal and optic nerve injuries, which are challenging to treat and can lead to permanent vision loss and will have huge impact on public health system in long-term perspective.

It is also important to note that, in addition to trauma-induced injuries, the study identified ocular changes associated with the aging process, including drusen, retinal striae, vascular disorders, and macular and pigmentary abnormalities. These age-related changes, commonly observed in individuals over 45 years of age, can complicate the diagnosis and treatment of ocular trauma within the context of armed conflict. Vascular disorders, in particular, may be related to systemic conditions such as hypertension and diabetes. The study focused on fundoscopic images, which do not capture damage to the lens, cornea, conjunctiva, or anterior chamber, thus limiting the scope to retinal changes.

This disparity could be attributed to various factors, including the nature of the injuries sustained by military personnel, who are more exposed to intense combat-related trauma such as fragmentation and blast injuries, which are more likely to cause severe retinal and macular damage (32). Conversely, civilians, while also exposed to war-related injuries, may face different patterns of injury due to the nature of the conflict they are involved in, such as indirect exposure to blasts or shrapnel, which may result in different injury profiles (32).

The type of hospital to which military personnel are referred—military or civilian—can significantly influence the diagnosis and treatment of eye injuries. Military hospitals, equipped to manage severe trauma such as fragmentation and blast-related injuries typical in combat, often adopt specialized treatment strategies. In contrast, civilian hospitals may employ a more general approach to trauma care. This distinction in medical facilities can affect both the reported severity and the treatment of ocular injuries. Therefore, when comparing civilians and military personnel, it is essential to consider differences in medical care and referral systems, as these factors impact injury management and long-term outcomes. Such considerations are crucial for accurately interpreting study results on war-related ocular trauma.

A limitation of this study is the difficult access to specialized medical care in war zones and, consequently, the limited availability of complete medical records. An undoubted limitation was the fact that the study was single-center by which the results obtained cannot be fully generalized.

Future research should prioritize enhancing diagnostic tools and therapeutic approaches for war-related eye injuries, focusing on faster diagnosis and improved access to specialized care in conflict zones. Prompt medical attention significantly improves recovery outcomes and reduces complications, but delays in diagnosis and limited access to appropriate facilities in danger zones increase the risk of permanent vision loss. Developing technologies for quicker and more accurate detection, coupled with better healthcare organization in conflict areas, is essential for early intervention and improved treatment outcomes.

## 5 Conclusions

This study provides a detailed analysis of eye injuries in the Ukrainian population due to the ongoing conflict, with a focus on macular disorders, retinal vascular changes, and optic nerve disorders, which were the most prevalent. The high rate of eye injuries in military personnel underscores the importance of timely treatment to prevent long-term vision loss. Future research should focus on improving diagnostic tools and therapeutic approaches for war-related ocular trauma. This study provides a detailed analysis of the type and type of eye injuries in the Ukrainian population caused by the ongoing armed conflict, with mechnical damage affecting macular disorders, retinal vascular lesions and optic nerve disorders, among others, which were the most common types of eye injuries. The high rate of eye injuries in both civilians and military personnel underscores the importance of prompt treatment to prevent long-term vision loss. The paper provides information for a specific conflict area, and the results can be used to implement changes at other clinical centers in the conflict zone. The results can help redistribute specialized equipment and supplement specialized apparatus.

## Data Availability

The raw data supporting the conclusions of this article will be made available by the authors, without undue reservation.
